# Melatonin in Male Dromedary Camel (*Camelus dromedarius*) Seminal Plasma and Its Specific MT1 and MT2 Receptors on Sperm Membranes

**DOI:** 10.3390/ani15010083

**Published:** 2025-01-02

**Authors:** Lamia Doghbri, Melissa Carvajal-Serna, Moufida Atigui, Adriana Casao, Victoria Peña-Delgado, Mabrouk-Mouldi Seddik, Mohamed Dbara, Rosaura Pérez-Pé, Mohamed Hammadi

**Affiliations:** 1 Livestock and Wildlife Laboratory (LR16IRA04), Arid Lands Institute, University of Gabès, Médenine 4119, Tunisia; lamia.doghbri@gmail.com (L.D.); atigui2009@gmail.com (M.A.); mabrouk.seddik16@gmail.com (M.-M.S.); mohamed.dbara@gmail.com (M.D.); 2 Faculty of Sciences of Gabès, University of Gabès, Erriadh, Gabès 6072, Tunisia; 3 Grupo BIOFITER, Instituto Universitario de Investigación en Ciencias Ambientales de Aragón (IUCA), Facultad de Veterinaria, Universidad de Zaragoza, Miguel Servet 177, 50013 Zaragoza, Spain; mcarvajals@unal.edu.co (M.C.-S.); adriana@unizar.es (A.C.); vpdelgado@unizar.es (V.P.-D.); rosperez@unizar.es (R.P.-P.); 4 Institution for Agricultural Research and Higher Education (IRESA), 30 Rue Alain Savary Tunis Belvédère, Tunis 1002, Tunisia

**Keywords:** camel, spermatozoa, testosterone, humidity, melatonin membrane receptors

## Abstract

Camels (*Camelus dromedarius*) are seasonal short-day breeders, regulated by photoperiod and melatonin secretion. However, no studies have explored melatonin levels in camel seminal plasma or their relationship with testosterone, age, or climatic factors, nor is it known whether melatonin receptors exist in camel spermatozoa to respond to seminal melatonin. This study aimed to analyze melatonin levels in camel seminal plasma and its specific receptors in spermatozoa. Semen samples were obtained from November to March (breeding season). Testosterone and melatonin levels were measured in seminal plasma by ELISA. Melatonin receptors were localized in spermatozoa using immunofluorescence, and their presence was confirmed by Western Blot. Melatonin levels were higher from November to January and decreased in February and March. No correlation between testosterone and melatonin levels was found, but both hormones were negatively correlated with daylength (*p* = 0.0089 and *p* = 0.0688, respectively). Testosterone, but not melatonin, levels were affected by age. Two melatonin receptors (MT1, MT2) were detected on camel spermatozoa, with several immunotypes labeled mainly in the tail and post-acrosome region, but also in the acrosome and neck. Western Blot analysis confirmed the presence of these receptors, showing a 39 kDa band for MT1 and a 36 kDa band for MT2. Understanding melatonin’s effects on sperm could help ejaculates’ processing procedures, semen handling, and infertility issues in camels.

## Simple Summary

This study analyzes melatonin levels in dromedary camel seminal plasma and characterizes the presence of melatonin receptors (MT1 and MT2) on sperm membranes. Melatonin levels were found to be highest during the shorter days of the breeding season, though no direct link with testosterone levels was observed. Additionally, two melatonin receptors, MT1 and MT2, were identified on camel sperm, primarily located in the tail and post-acrosome regions. These findings are interesting and pave the way for future studies on the role of melatonin receptors in the physiology of dromedary camel semen.

## 1. Introduction

Camels (*Camelus dromedarius*) are traditionally bred on pasture in herds of 40 to 80 females and a single mature male in extensive or semi-intensive systems [1]. Dromedaries are considered seasonal short-day breeders. In Tunisia, the breeding season or “rut” lasts from December to March [1] and seasonality is one of the main factors affecting reproductive performance.

The camel breeding season spans a time period characterized by a fluctuation of the photoperiod. Like many other species, camels respond to these photoperiod variations with significant changes in their melatonin secretory pattern. Melatonin is produced by the pinealocytes of the pineal gland during the night [2] and is then released into the peripheral blood, where it has been shown to affect testicular activity [3]. It can, however, be produced locally in many organs, including testicular cells [4]. Melatonin secretion modulates the hypothalamic-pituitary-reproductive axis, affecting the pulsatile secretion of the gonadotrophin-releasing hormone (GnRH), thus the release of LH and FSH from the pituitary gland, and therefore increasing the testosterone levels secreted by the testis [5]. The secreted testosterone is metabolized by the enzyme aromatase to estrogens [6], which seems to control sperm motility [7]. However, in camels, these hormonal changes have not only been associated with variation in the photoperiod but also with ambient temperature; thus, better quality semen is collected during December and January when the temperature is lower and the daylength is shorter [8].

As demonstrated by Swelum et al. [9], the use of melatonin implants in dromedary males during the non-breeding season improved their reproductive performance and increased testes and scrotum size, libido, mating ability, serum melatonin and testosterone levels, and semen quality, especially sperm progressive motility and viability. The effects of exogenous melatonin could be explained by modulation of the hypothalamic-pituitary-gonadal axis [10,11,12]. However, the enhancing effect of exogenous melatonin in dromedary bull semen quality can also be explained by a direct action of this hormone on the spermatozoa. Melatonin has a broad range of biological roles in the cells: it is a free-radical scavenger [13], an anti-apoptotic molecule, an immunomodulator, a biological modulator of the circadian rhythms. Many of these effects, including the signaling of the seasonality, are mediated by its interactions with the melatonin receptors MT1 and MT2. These receptors, previously known as Mel1a and Mel1b receptors, belong to the G-protein-coupled receptor (GPCR) superfamily [14]. Melatonin receptors are located along the hypothalamic-pituitary-testicular axis in many cellular types [4]. Furthermore, the immunolocalization of MT1 and MT2 receptors on spermatozoa has been demonstrated in many mammals, such as ram [15,16], donkey, boar, stallion, dog, bull and red deer [17]. However, to the best of our knowledge, no study has described the presence of melatonin receptors in camel spermatozoa.

Thus, the objectives of this study were: (1) to investigate possible variations in melatonin and testosterone levels in camel seminal plasma and (2) to detect the presence of MT1 and MT2 melatonin receptors in camel spermatozoa.

## 2. Materials and Methods

### 2.1. Animals and Location

Six clinically healthy male dromedary camels (*Camelus dromedarius*, Age: 6–15 years, BW (body weight): 514–675 kg and body condition score: 4.0 ± 0.4 AU (range from 0 to 5 [18]). Three animals in group 1, aged 6–8 years with BW 589.75 ± 44.08 kg, and 3 animals in group 2, aged 13–15 years with BW 603.55 ± 35.66 kg, were used for this study during three breeding seasons: November 2017–March 2018, November 2018–March 2019 and November 2019–March 2020. The camels were reared under an intensive system at the Arid Lands Institute’s Artificial Insemination Centre in Medenine, Tunisia (33°30′ N, 10°40′ E). They were housed individually in a single box (height = 3 m, length = 5 m and width = 3 m) with a sand floor. The sires were fed with 4.5 kg of oat hay at 9:00 a.m., and 4 kg of a mixture of barley, wheat bran, olive cake, minerals and vitamins at 3:00 p.m. Water was available once every 2 days.

### 2.2. Semen Collection and Seminal Plasma Preparation

Semen was collected twice a week using a bovine artificial vagina (IMV, France) with a female camel maintained in a couched position, following the standardized procedure suggested by Padalino et al. [19]. The ejaculate was then evaluated for volume, mass motility, sperm viability, and concentration as part of routine analyses [20,21] to assess sperm quality. After the semen analysis, only ejaculates with motility ≥ 4 (scale 0–5) and viability > 60% were selected for melatonin and testosterone analysis, immunolocalization of melatonin receptors and protein semen extraction for western blot.

A total of 118 ejaculates, distributed across the three study seasons (season 1:36, season 2:49 and season 3:33 ejaculates), were used to determine testosterone and melatonin concentrations in camel seminal plasma. After collection, ejaculates were naturally liquefied during two hours at 36 °C in water bath (memmert^®^, type WNB14, Schwabach FRG, Germany). Then, 2 mL of the ejaculate were centrifuged at 2800× *g* for 30 min at 4 °C, the pellet was discarded, and the obtained seminal plasma was stored at −20 °C until further assays. On the other hand, eighteen liquefied ejaculates (group 1:10 and group 2:8 ejaculates), obtained in February and March 2020, were used to assess the localization of melatonin MT1 and MT2 receptors in camel spermatozoa. Four slides (two slides for each receptor) were prepared from each ejaculate.

### 2.3. Climatic Parameters

The daylength data were collected from the website https://www.timeanddate.com/ (accessed on 2 February 2023). An automatic weather station (CR510 Campbell Scientific data logger, Logan, UT, USA) provided the average ambient temperature and humidity data throughout the study. The meteorological data are summarized in Table 1.

### 2.4. Hormone Analyses

#### 2.4.1. Melatonin in Seminal Plasma

Melatonin concentration in camel seminal plasma were determined using a commercial competitive immunoassay (Direct saliva melatonin ELISA kit, Bühlmann Laboratories AG, Schönenbuch, Switzerland, sensitivity: 0.5 pg/mL, intra-assay variability: 5.2%, inter-assay variability: 10.2%), respecting the manufacturer’s instructions. iIn brief, 100 μL of each sample, both control and calibrators were added in duplicate to a microtiter plate coated with an anti-melatonin antibody and incubated for 16 h at 4 °C. Then, 50 μL of biotinylated melatonin were dispensed to each well and incubated for 3 h at 4 °C. After three washes, 100 μL of streptavidin conjugated to horseradish peroxidase (HRP) were added to the wells and incubated for an additional 60 min in an orbital shaker set at 600 rpm at room temperature. The wells were rinsed three times and 100 μL of TMB substrate were applied to each well and incubated for 30 min at 600 rpm, protected from direct light. When incubation ended, 100 μL of 0.25 M H_2_SO_4_ solution were added, and absorbance was measured on a microtiter plate reader (TECAN Spectrafluor plus, Zurich, Switzerland) at 450 nm. All samples were loaded in duplicate and analyzed in the same assay.

#### 2.4.2. Testosterone in Seminal Plasma

Testosterone evaluation in camel seminal plasma was performed using a total testosterone commercial ELISA kit assay (Testosterone-ELISA KAPD1559, DIAsourceImmunoAssay S.A., Louvain-la-Neuve, Belgium. Sensitivity: 0.05 ng/mL, intra-assay variability: 4.8%, inter-assay variability: 7.1%), following the manufacturer’s instructions. Fifty μL of each sample, both control and calibrators, followed by 100 μL of testosterone labelled with horseradish peroxidase (HRP), were loaded in duplicate in a microtiter plate coated with an anti-testosterone specific antibody and incubated for 1 h at room temperature (25 °C). After incubation, the wells were washed three times, and 100 μL of 3,3′,5,5′-tetramethylbenzidine substrate (TMB) were added to each well. Then the plate was protected from direct light and incubated for 30 min at room temperature. After incubation, 100 μL of stop solution (0.2 M HCl) were added, and absorbance was measured on a microtiter plate reader (TECAN Spectrafluor Plus, Zurich, Switzerland) at 450 nm. All samples were loaded in duplicate and analyzed in the same experiment.

### 2.5. Immunofluorescence Assay

Melatonin MT1 and MT2 receptors in camel spermatozoa were localized and identified by the immunofluorescence assay as described by Casao et al. [15], with some modifications. Shortly, aliquots of 4 × 10^6^ spermatozoa were fixed with 3.7% formaldehyde (*v*/*v*) in phosphate-buffered saline (PBS: 137 mM NaCl, 2.7 mM KCl, 8.1 mM Na_2_HPO_4_, and 1.76 mM KH_2_PO_4_, pH = 7.2) for 20 min at room temperature. The cells were then centrifuged for 6 min at 900× *g* at room temperature, and the pellet was re-suspended in 500 μL of PBS. Subsequently, 40 μL of cell suspension was spreadonto poly-L-lysine-coated slides and left at room temperature for 3 h to facilitate properadhesion of the spermatozoa to the slide. The slides were kept at 4 °C until transportation to the Biochemistry and Molecular and Cellular Biology laboratory at the Veterinary Faculty of the University of Zaragoza, Spain, for the immunofluorescence assay.

Before washing the slides three times for 5 min with PBS, another 50 µL of poly-L-lysine were added to ensure adequate spermatozoa adhesion during the immunostaining procedure. Then, the non-specific binding sites were blocked with 5% BSA (*w*/*v*) in PBS for 4 h at room temperature in a wet chamber. Following three additional PBS washes, the spermatozoa were incubated with the primary antibody for melatonin receptor MT1 (MTNR1A mouse polyclonal antibody; Abnova, Taipei, Taiwan; Cat# H00004543-A01, RRID: AB_462681) diluted 1:10 in PBS with 1% BSA, or the primary antibody for melatonin receptor MT2 (MTNR1B Rabbit Polyclonal Antibody, Acris Antibodies GmbH, Herford, Germany; Cat# AP01322PU-N, RRID: AB_1619198) diluted 1:20 in PBS with 1% BSA overnight at 4 °C in a wet chamber.

The next day, after performing three PBS washes, the cells were exposed to the secondary antibodies: Alexa Fluor 594 chicken anti-mouse (Cat#A-21201, RRID: AB_2535787) for the MT1 receptor and Alexa Fluor 488 chicken anti-rabbit (Cat# A-21441, RRID: AB_2535859) for the MT2 receptor (Thermo Fisher Scientific, Waltham, MA, USA), both diluted 1:400 in PBS containing 1% BSA (*w*/*v*) for 1 h at room temperature in a dark humidified chamber.

The slides were then washed three times with PBS. For the MT1 slides, Hoechst staining (33,342, trihydrochloride, Life Technologies, Carlsbad, CA, USA) was added as a cellular marker to visualize the sperm cells. After drying, the immunofluorescence was visualized using a Nikon Eclipse E-600 microscope (Tokyo, Japan) under epifluorescence illumination.

### 2.6. Western Blotting

Sperm membrane proteins were extracted by incubation of 8 × 10^6^ cells in 100 μL extraction buffer (1 M TRIS-HCl, 4% (*w*/*v*) sodium dodecyl sulphate 10% (*v*/*v*)) at 100 °C for 5 min in a sand bath. After a centrifugation at 13,000× *g* for 5 min, the supernatant was recovered, 5% (*v*/*v*) glycerol 20%, 5% β-mercaptoethanol and protease inhibitor cocktail (SigmaAldrich Corporation, St. Louis, MO, USA) were added, and the protein samples were stored at −20 °C until their use.

For sodium dodecyl sulfate-polyacrylamide gel electrophoresis (SDS-PAGE), extracted protein (a mix of sperm proteins from 6 different males) were concentrated 7 times with Amicon Ultra 10 K device (Sigma Aldrich Corporation, St. Louis, MO, USA). Ten µL of the concentrated proteins were loaded after adding 0.02% (*v*/*v*) bromophenol blue on 12% and 10% (*w*/*v*) acrylamide gels for the MT1 and MT2 receptors, respectively. The proteins were separated by standard electrophoresis and transferred to a polyvinylidene difluoride (PVDF) membrane with the Trans-Blot Turbo Transfer System (Bio-Rad Laboratories, Hercules, CA, USA).

After rinsing the membrane with ultrapure water for 2 min in an orbital shaker (100 rpm), the membrane was treated with the antigen pre-treatment solution SuperSignal^®^ Western Blot Enhancer, (ThermoFisher Scientific, Waltham, MA, USA) according to the manufacturer’s instructions. After the blocking of non-specific sites on the membrane with SuperBlock Blocking Buffer (ThermoFisher Scientific, Waltham, MA, USA) for 10 min with shaking, the melatonin receptors were immunodetected by incubating for 1 h at room temperature with the primary antibody Mel-1A-R rabbit polyclonal antibody against the MT1 receptor (GeneTex Inc., Irvine, CA, USA; Cat# GTX100003, RRID: AB_1241048) or rabbit polyclonal antibody against the MT2 receptor (Acris Antibodies, GmbH, Herford, Germany; Cat# AP01322PU-N, RRID: AB_1619198), both diluted 1:500 in the Primary Antibody Diluent (ThermoFisher Scientific, Waltham, MA, USA). The membranes were washed 4 times in 0.05% (*v*/*v*) Tween-20^®^ PBS and then incubated with a donkey anti-rabbit IRDye 800CW (LI-COR Biosciences, Lincoln, NE, USA; Cat# 926-32212, RRID: AB_621848) secondary antibody, diluted 1:7500 (*v*/*v*) in the SuperBlock Blocking Buffer, for 30 min at room temperature. After washing, the membranes were scanned, and the bands were identified using the Odyssey CLx Imaging System and the Image Studio Acquisition Software (LI-COR Biosciences, Lincoln, NE, USA, https://www.licor.com/bio/image-studio/, accessed on 21 October 2024). Precision Plus Protein™ Standards (BIO-RAD, Catalog #161-0374), covering a range of 10–250 kDa, were used as molecular weight markers, allowing precise estimation of the receptor sizes. Extracted proteins from ram spermatozoa were used as a positive control [15].

### 2.7. Statistical Analyses

Statistical analyses were performed using Statistical Analysis Systems software (SAS software 2012, version 9.4 (SAS Institute, Cary, NC, USA)). For statistical purposes, the camels were classified into two groups: group 1 (3 sires) aged 8 years and group 2 (3 sires) aged 15 years, at the end of this study to assess the effect of camel age on melatonin and testosterone levels in the seminal plasma and on the localization of melatonin receptors (MT1 and MT2) in the spermatozoa.

Melatonin and testosterone concentrations in the seminal plasma were analyzed using the PROC mixed model. Normality was verified using the Kolmogorov-Smirnov test, and the melatonin concentration was transformed by the Napierian logarithm function. Then, the homogeneity of variances was verified using the Bartlett test and linearity was confirmed. The PROC mixed model included the general mean, age group of sires, month and breeding season of semen collection as the fixed variables, while sire identity and residual error were included as random variables. Except for the seminal plasma melatonin concentration, which is presented as ln-transformed least squares means (LSM) ± standard errors (SE), all the given estimates are presented as least squares mean (LSM) ± standard errors (SE).

One-way ANOVA was used to compare monthly mean daylength, temperature, and humidity between the three breeding seasons. Newman-Keuls Multiple Comparison Test was used to detect difference between months and seasons. Pearson correlations between the melatonin and testosterone levels in seminal plasma and the climatic parameters were determined using PROC CORR of SAS (2012). *p*-values are considered significant at <0.05 and tendencies at <0.08.

Differences in the distribution of the melatonin receptors between the age groups were evaluated using the COMPPROP multiple comparisons of proportions procedure for a 2 × 6 and a 2 × 7 contingency table analysis for MT1 and MT2, respectively**.**

## 3. Results

### 3.1. Melatonin and Testosterone Concentrations in Seminal Plasma

**Figure 1:**
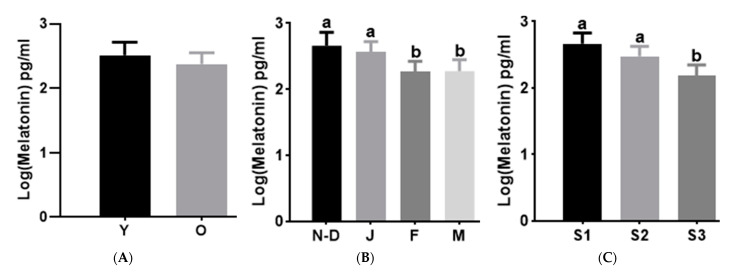
Effects of age (**A**), month (**B**) and season of collection (**C**) on Log (melatonin) in camel seminal plasma. Values are shown as LSM ± SE of *n* =118. a, b indicates *p* < 0.05. Y: Young males; O: Old males; N–D: November–December; J: January; F: February; M: March; S1: Season 1 (2017–2018); S2: Season 2 (2018–2019); S3: Season 3 (2019–2020).

The melatonin concentration in camel seminal plasma averaged 15.4 ± 1.7 pg/mL. Log (melatonin) was not affected by the age of the males (*p* = 0.61, Figure 1A). However, it was affected by the month of semen collection, with the maximum values observed in November–December and January (Figure 1B) and the lowest in February and March (*p* = 0.06). When comparing the log (melatonin) concentration in the breeding seasons of different years, the lowest value was observed in breeding season 3 (*p* < 0.01) (Figure 1C).

**Figure 2:**
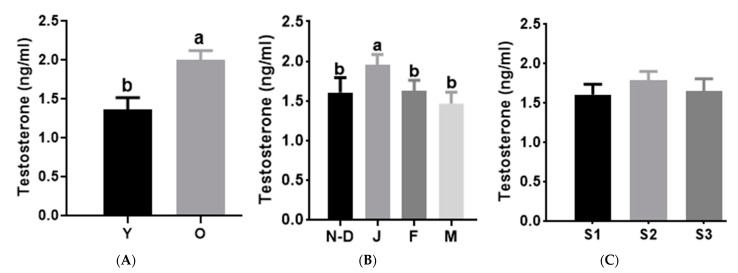
Effects of age (**A**), month (**B**) and season of collection (**C**) on testosterone in camel seminal plasma. Values are shown as LSM ± SD of *n* = 118. a, b indicates *p* < 0.05. Y: Young males; O: Old males; N–D: November–December; J: January; F: February; M: March; S1: Season 1 (2017–2018); S2: Season 2 (2018–2019); S3: Season 3 (2019–2020).

The testosterone concentration in camel seminal plasma averaged 1.9 ± 0.1 ng/mL. It was higher in the old camels group than in the young camels one (*p* < 0.001, Figure 2A) and reached its peak in January and decreased in March (*p* < 0.05, Figure 2B). The testosterone did not vary between breeding seasons of different years (*p* = 0.33). There was no correlation between melatonin and testosterone levels in seminal plasma.

### 3.2. Correlations Between Hormone Levels in Seminal Plasma and Climatic Parameters

Pearson correlations between hormones in the seminal plasma and the climatic parameters are presented in Table 2. Testosterone and melatonin in the seminal plasma were negatively correlated with the daylength (*p* = 0.0089, *p* = 0.0688, respectively) but positively correlated with the humidity (*p* = 0.07, *p* = 0.06, respectively). No correlation with temperature was observed.

### 3.3. MT1 and MT2 Melatonin Receptors in Spermatozoa

**Figure 3:**
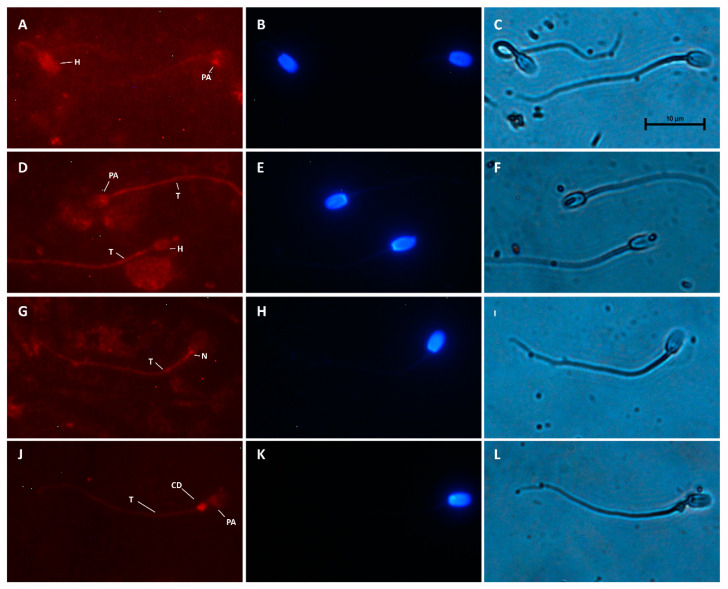
Distribution of MT1 melatonin receptors in camel spermatozoa, evaluated by the indirect immunofluorescence method. Immunostaining in the head (H), post-acrosome (PA), neck (N), tail (T) and cytoplasmic droplet (CD) was evidenced. Magnification 1000×. MT1 receptors (**A**,**D**,**G**,**J**), Hoechst staining (**B**,**E**,**H**,**K**), and bright field (**C**,**F**,**I**,**L**) are shown.

Both MT1 and MT2 receptors were detected in camel spermatozoa by the immunofluorescence assay and several immunotypes for each receptor were identified. The distribution of MT1 and MT2 immunotypes on the spermatozoa varied significantly (*p* < 0.05). MT1 was primarily located at the tail (44.30% of sperm cells) (Figure 3D,G,J) and post-acrosome region (23.40% of sperm cells) (Figure 3A,D,J). MT1 was also identified all over the head (Figure 3A,D), with or without labelling in the neck and tail (Figure 3D and 3A, respectively). Spermatozoa with only neck and tail labelling were also found (Figure 3G). When present, the cytoplasmic droplet also showed intense staining (Figure 3J) (4.43% of spermatozoa).

**Figure 4:**
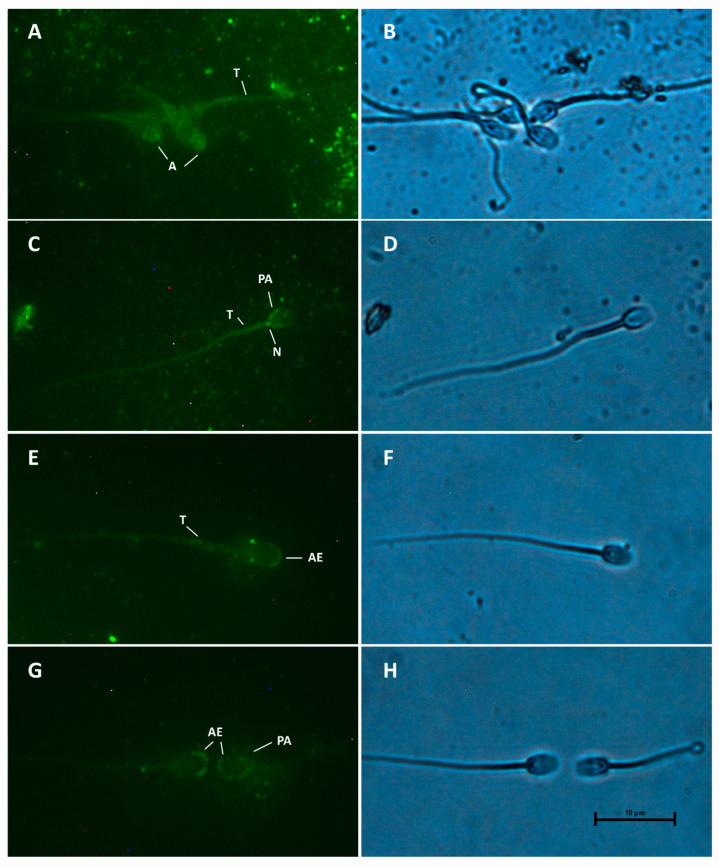
Distribution of MT2 melatonin receptors in camel spermatozoa, evaluated by the indirect immunoassay method. Immunostaining in head (H), acrosome (A), post-acrosome (PA), neck (N), tail (T) and apical edge (AE) was evidenced. Magnification 1000×. MT2 receptors (**A**,**C**,**E**,**G**) and bright field (**B**,**D**,**F**,**H**) are shown.

MT2 receptors were also mainly located at the post-acrosome region (Figure 4C) and tail (Figure 4A,C,E), (31.37% and 25.26%, respectively). However, some of them showed a bright signal at the acrosomal region (Figure 4A), the apical edge (Figure 4E,G), or both the apical edge and the post-acrosome region (Figure 4G). The post-acrosomal reactivity was also related to staining in the tail and neck, with or without apical edge reactivity (Figure 4C).

**Figure 5:**
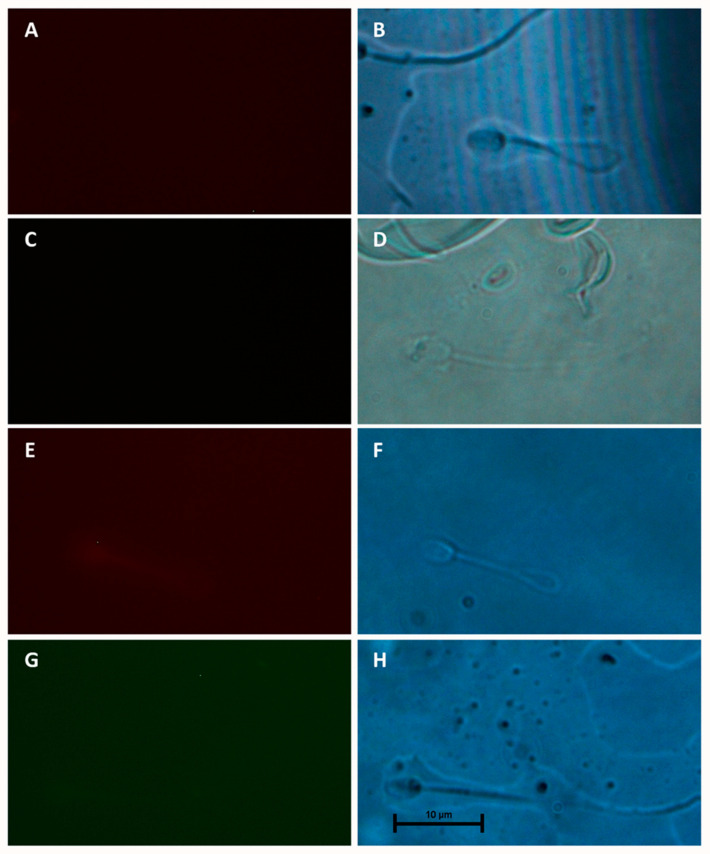
Indirect immunofluorescence controls. Samples were incubated with only the MT1 (**A**) or MT2 (**C**) primary or secondary antibody (**E**) and (**G**) for Alexa Fluor 594 anti-mouse antibody and Alexa Fluor 488 anti-rabbit antibody, respectively). Magnification 1000×. Fluorescence after 30 s exposition (**A**,**C**,**E**,**G**) and bright field (**B**,**D**,**F**,**H**) are shown.

The omission of the primary (Figure 5A and 5C for MT1 and MT2, respectively) or secondary antibody (Figure 5E and 5G for MT1 and MT2, respectively) resulted in no fluorescent signal.

**Figure 6:**
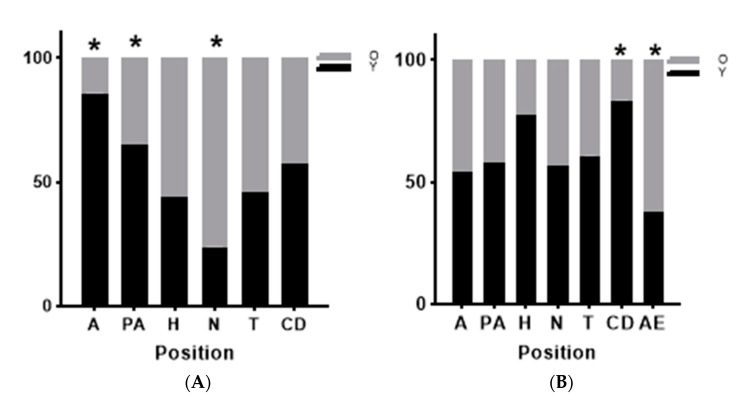
Comparison among age classes of males at different melatonin receptors localizations (MT1 (**A**) and MT2 (**B**)) in acrosome (A), post-acrosome (PA), head (H), neck (N), tail (T), cytoplasmic droplet (CD) and apical edge (AE). Values are shown as percentage of localization of *n* = 439 spermatozoa. * indicates *p* < 0.05.

The distribution of MT1 (Figure 6A) receptors in spermatozoa varied according to camel age (X^2^ = 13.48, *p* = 0.0192). Older sires had significantly more MT1 receptors located in the neck region (76.2% of spermatozoa with neck labelling were from older camels), while younger camels had significantly more MT1 receptors at acrosomal and post-acrosomal regions, (85.7% and 64.8% of the MT1 receptors located there were detected in young males). As for the head, tail and cytoplasmic droplets, there was no effect of the age on these MT1 receptor localizations.

MT2 receptors were predominant at the post-acrosomal region in both young and old sires (32.2% and 30.3%, respectively) followed by the tail (27.3% and 22.6%, respectively) with no significant differences between ages (Figure 6B). However, older camels had significantly (X^2^ = 16.69, *p* = 0.01) more spermatozoa with MT2 receptors located at the apical edge compared to younger ones (20.7% and 9.7%, respectively). Although MT2 receptors on cytoplasmic droplets were rarely observed (1.3%), they were more frequent in young sires (83.3% of the MT2 receptors located on cytoplasmic droplets were identified in young camels).

### 3.4. Melatonin Receptors Identification by Western Blot

**Figure 7:**
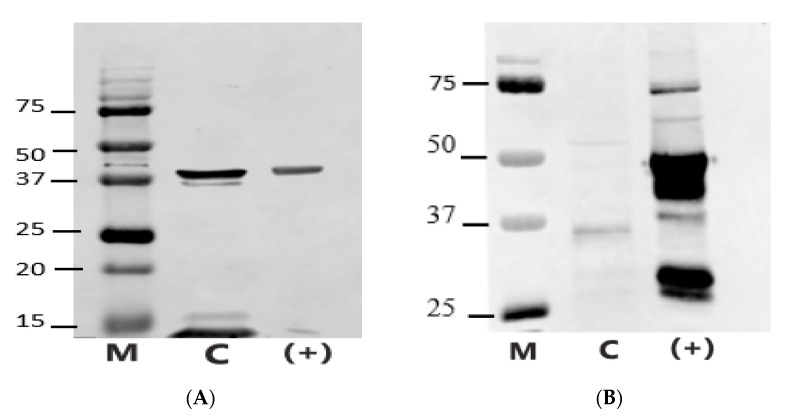
Representative Western blot images showing the presence of MT1 (**A**) and MT2 (**B**) melatonin receptors in protein extracts from camel spermatozoa (M: Molecular weight marker (kDa), C: Camel sperm proteins, (+): Positive ram control).

Western blot analysis of proteins extracted from camel spermatozoa revealed a 39-kDa band, consistent with the MT1 receptor (Figure 7A), which matched the band observed in the positive control (ram sperm proteins).

For the MT2 receptor (Figure 7B), a band at approximately 36 kDa was detected, slightly smaller than the 39 kDa band observed in the positive control. Additionally, a faint band around 55 kDa was observed in the MT2 camel profile.

## 4. Discussion

Camels are seasonal breeders. Previous studies have demonstrated the relative importance of the environmental factors, as well as the neuroendocrine mechanisms underlying seasonality in camel species [5,22,23]. Seasonal variations of melatonin concentration in blood have also been previously described in camels [5] with higher levels during the breeding season in dromedary bulls [9,24]. In this study, melatonin levels were measured, for the first time, in the seminal plasma of dromedary camels, being higher from November to January, when the night duration was longer. Although melatonin could be produced locally by testicular cells [4], these findings suggest that the melatonin in camel seminal plasma may primarily originate from the pineal gland; however, this hypothesis remains to be explored. In our study, melatonin levels in seminal plasma were not affected by the age of camel bulls. However, El-Allali et al. [5] revealed an age effect on blood plasma melatonin, with lower levels in old camels due to an age-dependent decrease in pineal melatonin synthesis. The absence of an age effect in this study may be attributed to the fact that the males were neither pre-pubertal nor of advanced age. Daylight exposure and photoperiod changes are the main regulators of melatonin production [25], although climatic conditions, including temperature and humidity, could influence its release in vertebrates [26]. During this study, plasma melatonin was significantly lower in the third breeding season than in the first and second seasons. We detected a positive correlation between seminal plasma melatonin and humidity, but not temperature, and a negative correlation with daylength. A previous study conducted on mature male dromedaries had shown a positive correlation between sexual behavior and evening humidity, and a negative correlation with temperature [27]. As short-day breeders, the negative correlation between melatonin and testosterone with daylength in camel seminal plasma is theoretically attributed to longer dark periods activating the pineal gland, which then stimulates the hypothalamic-pituitary-gonadal axis, leading to the secretion of hormones such as testosterone [5].

The testosterone concentrations in the seminal plasma and blood of dromedary camels are used as fertility-associated biomarkers [28]. Age is an essential aspect when considering the potential fertility of a camel bull [29]. In the present study, the seminal plasma testosterone levels were higher in older camels. This difference may be explained by an increase in size and activity of Leydig cells with age, along with the growth in number and size of Sertoli cells, which are essential for supporting sperm production and fertility [30]. These age-related variations in testosterone concentration were previously described in camel blood [23,29,30] with a significant increase in old males (from 5 up to 13 years old) compared with young ones (up to 4 years old).

Moreover, the seminal plasma testosterone concentration found in the present study peaked in January and decreased in March. The rise during the breeding season, from November-December to January, may be attributed to the increase in the volume and number of Leydig cells during the rutting period due to the increase in blood plasma testosterone concentration [30,31,32]. Moreover, this testosterone augmentation could be due to the increased sensitivity of Leydig cells to LH or the enhanced secretion of LH from the pituitary gland due to the stimulatory effect of melatonin on the hypothalamus-pituitary axis [5], or both [33,34]. The high circulating testosterone levels in the mating season are responsible for morphological and histological changes in the testis and the augmentation of the camel sire’s libido and typical sexual behavior patterns [35,36,37,38,39].

Although melatonin has been shown to promote testosterone synthesis in Leydig cells of various species, including increased testosterone levels in animal models such as sheep and mice [40], our results in dromedaries did not reveal a direct correlation between melatonin and testosterone concentrations in seminal plasma. This might be hypothesized that melatonin’s regulatory mechanisms on testosterone might be species-specific or could differ in the reproductive physiology of dromedaries compared to other mammals.

These observed variations in melatonin levels in camel seminal plasma could affect the spermatozoa in contact with it. Several studies in other species have described direct effects of melatonin on spermatozoa [41,42]. Some of these actions can be exerted by binding to specific receptors present in the sperm membrane. These receptors have been described in several species, including human, hamster [43], ram [15], goat [44] donkey, stallion, boar, bull and dog [17].

Based on genetic data give in the databases, Gao et al. [45] reconstituted the MT1 and MT2 receptors’ phylogenetic tree in several mammalian species, including camels. Nevertheless, to our knowledge, this is the first study showing the existence of melatonin receptors on spermatozoa of dromedary bulls. According to our indirect immunofluorescence results, dromedary spermatozoa are characterized by high variability in the distribution of both types of melatonin receptors compared to those reported by Gonzalez-Arto et al. [17], Casao et al. [15] and Cardenas-Padilla et al. [44] in other species.

MT1 is distributed in the head, neck, tail, acrosome, and post-acrosome of dromedary spermatozoa, while MT2 is localized in the acrosome, neck, apical edge, and tail. This distribution aligns with findings in rams [15] and various other species like bull and donkey [17], although dromedaries show a broader receptor distribution, particularly for MT1, also detected in the cytoplasmic droplet, as seen in stallions [17]. Compared to goats [44], dromedary sperm exhibits a more diffuse distribution, especially for MT2, which extends beyond the neck to the apical edge and tail. In our study, the variability in MT1 and MT2 receptor distribution could be attributed to the high heterogeneity of the ejaculated semen. In fact, the physiological status of the spermatozoa could influence the distribution and density of melatonin receptors, since the maturation degree, capacitation and apoptosis processes involved membrane changes that may affect their distribution [46,47]. A morphological analysis could provide more insights into the sources of variability in the distribution of melatonin receptors in camel spermatozoa.

MT1 and MT2 receptors were identified mainly in the post-acrosomal and flagellum regions of the camel spermatozoa, but other locations were also detected. The presence of different immunotypes in the same ejaculate suggests that the function of these receptors may vary. They could be implicated in the anti-apoptotic activity or the anti-oxidant defense of the spermatozoa. In fact, the dromedary camel is a non-spontaneous ovulatory species. Ovulation occurs following coitus during mating, triggered by a systemic action of the seminal male plasma through nerve growth factor beta (β-NGF) ([1,48,49], and fertilization takes place relatively late compared to other ruminants. Ovulation takes about 36–72 h post-mating to occur [50], which implies a possible need for additional protection of the spermatozoa against membrane degradation and DNA fragmentation. Moreover, the MT1 receptor has been reported to be involved in apoptosis protection in human spermatozoa [42,51,52]. On the other hand, due to the melatonin receptors being located in the acrosome, post-acrosome and all over the head, they can also be involved in the fertilization process [53]. MT2 receptors were also identified at the apical edge of some camel spermatozoa, which could also suggest the possible involvement of MT2 receptors in the capacitation process in this species, as it has been described in others as ovine [17].

We have detected that MT1 receptors were predominant in the camel spermatozoa tail (44.3% of the detected MT1 receptors), indicating a possible indirect role in the motility of the sperm, since melatonin can affect mitochondrial function to generate ATP, which is essential for the tail movement that drives motility in sperm cells [42], but we recognize that our work has not provided proof of this. In hamster sperm, MT1 rather than MT2 receptors are involved in the hyperactivation of spermatozoa during the penetration of the zona pellucida [54]. However, the co-expression of both melatonin receptor subtypes, particularly at the tail and post-acrosome locations, suggests a possible cross-regulation of both receptors, as already proposed for ovine brain tissues by Cogé et al. [55].

Our study also revealed an age effect on the distribution of MT1 and MT2 receptors. Younger camels had more MT1 receptors at the acrosomal and post-acrosomal regions, while older bulls had more MT1 receptors localized in the neck region. The results also showed that older camels had more MT2 receptors at the apical edge of the spermatozoa, while most of the MT2 receptors in cytoplasmic droplets were identified in young males. This aligns with previous findings on increased testicular reserves in older sires [29] and higher testosterone secretion [23,30], which may influence epididymal protein synthesis, crucial for sperm maturation [56]. An exploration of potential variations in sperm morphological defects between the two age groups may provide insights into the observed differences in receptor expression.

The Western blot results further underscore the species-specific variations. In dromedaries, we detected a 39 kDa band for MT1, consistent with the MT1 molecular weight also found in rams, donkeys, and other species spermatozoa [17]. However, the presence of an additional faint band below 39 kDa suggests possible isoforms or post-translational modifications unique to dromedaries. For MT2, a band just below 37 kDa and a faint band around 55 kDa were observed, likely indicating receptor dimerisation or interaction with other membrane proteins, such as G-proteins. This pattern contrasts with rams, with 75, 50–45 and 39 kDa bands [15]; goats, where MT2 bands appeared at 75, 42, and 35 kDa, and with boars and bulls, where MT2 was found at 28–45 kDa [17]. These molecular weight differences likely reflect species-specific variations in receptor amino acid sequence, dimerization and activation states, further emphasizing the diversity in melatonin receptor behavior across species.

## 5. Conclusions

In summary, this study has evaluated, for the first time, the concentration of melatonin in camel seminal plasma. We have also detected, for the first time, the presence of melatonin receptors MT1 and MT2 in camel spermatozoa. These findings suggest a possible role on melatonin in the sperms’ physiology, which should be investigated for applications related to semen processing or to infertility/subfertility therapies in this species. Although the reduced number of males represents a limitation in this work, we have been able to describe different immunotypes for MT1 and MT2, and detected significant differences in immunotype distribution with age. The considerable heterogeneity detected in the localization of receptors suggests a need for quantitative analysis of different immunotypes. Future research could explore the polymorphism of melatonin receptor genes and their potential link to semen quality and reproductive performance in dromedary bulls. This can help select suitable males for semen collection and artificial insemination programs in the future.

## Figures and Tables

**Table 1:** Mean daylength, temperature and humidity during the three breeding seasons of the study.

Season	Months	Daylength (h)	Temperature (°C)	Humidity (%)
S1 (2017–2018)	N–D	10.04 ± 0.05	14.23 ± 0.46 ^b^	61.92 ± 2.11 ^a^
	J	10.02 ± 0.01	13.60 ± 0.35 ^a^	69.43± 1.71 ^a^
	F	10.80 ± 0.06	12.8 ± 0.27 ^b^	68.80 ± 2.85 ^a^
	M	11.78 ± 0.07	18.0 ± 0.61 ^a^	56.08 ± 1.62 ^a^
	Mean	10.53 ± 0.77	14.75 ± 0.29 ^B^	62.58 ± 1.31 ^A^
S2 (2018–2019)	N–D	9.99 ± 0.04	15.15 ± 0.33 ^a^	59.49 ± 1.50 ^a^
	J	10.12 ± 0.02	10.97 ± 0.33 ^b^	68.84 ± 1.91 ^a^
	F	10.79 ± 0.06	12.22 ± 0.41 ^b^	66.12 ± 2.26 ^a^
	M	11.77 ± 0.06	15.92 ± 0.39 ^b^	62.47± 2.27 ^a^
	Mean	10.53 ± 0.76	14.75 ± 0.29 ^B^	63.10 ± 1.00 ^A^
S3 (2019–2020)	N–D	9.99 ± 0.04	15.61 ± 0.36 ^a^	52.61 ± 1.58 ^b^
	J	10.10 ± 0.03	13.97 ± 0.35 ^a^	59.28 ± 0.67 ^b^
	F	10.79 ± 0.06	14.8 ± 0.39 ^a^	59.36 ± 2.04 ^b^
	M	11.80 ± 0.06	15.86 ± 0.27 ^b^	46.68 ± 1.92 ^b^
	Mean	10.54 ± 0.06	15.24 ± 0.19 ^A^	54.03 ± 1.10 ^B^

S1: Season 1; S2: Season 2; S3: Season 3; N–D: November–December, J: January, F: February, M: March. ^a,b^: for each month between season, significant difference (*p* < 0.05) was indicated, ^A,B^: significant difference (*p* < 0.05) between seasons was indicated.

**Table 2:** Pearson correlations between melatonin, testosterone and climatic factors.

Parameters		Daylength	Temperature	Humidity
Melatonin	r	−0.174	−0.125	0.218
	P	0.0688	0.2935	0.0675
Testosterone	r	−0.256	−0.130	0.215
	P	0.0089	0.2873	0.0776

*n* = 110.

## Data Availability

The datasets generated during and/or analyzed during the current study are available from the corresponding author on reasonable request.
